# Phthisis Bulbi in a Retinitis Pigmentosa Patient after Argus II Implantation

**DOI:** 10.1155/2020/5608058

**Published:** 2020-08-17

**Authors:** Logan Vander Woude, Ramak Roohipour, Gibran Syed Khurshid

**Affiliations:** Department of Ophthalmology, University of Florida, USA

## Abstract

**Purpose:**

To report a previously unreported complication of phthisis after Argus II prosthesis implantation in a retinitis pigmentosa (RP) patient.

**Case:**

A 61-year-old male with advanced RP presented to the retina clinic. The patient had a history of vitrectomy in both eyes (OU) in Cuba in 1996. Pre-op visual acuity (VA) was no light perception (NLP) in the right eye and light perception (LP) in the left eye. The patient met the criteria for Argus II implantation and elected to proceed with surgery in his left eye in December 2017. The surgical implantation of the Argus II was successful without any complications. On postoperative day 1, his VA was stable at LP. He was satisfied with his ambulatory vision after the electrodes were turned on. Four months after surgery, the patient was complaining of aching pain; he was found to have preseptal cellulitis and was started on antibiotics. This swelling improved over two weeks, but when the patient returned, he had a two mm hyphema associated with mild ocular inflammation without an inciting event or reason on exam. The hyphema was treated and resolved after two weeks. However, one month after the hyphema resolved, at postoperative month six, the patient's vision in his left eye became NLP and began to demonstrate phthisical changes, including hypotony, Descemet membrane folds, and a vascular posterior capsular membrane. *Discussion*. The theoretical causes of phthisis bulbi after Argus II implantation include fibrous downgrowth, ciliary shut down due to immune reaction, inflammation, or trauma. While the cause of phthisis in this Argus patient is not certain and possibly multifactorial, it is important to note that phthisis is a possible complication of an Argus II implant, as this patient had no other obvious insult or reason for the phthisical change.

## 1. Introduction

Retinitis pigmentosa is an inherited retinal disease characterized by a progressive degeneration of rods, followed by cones and finally RPE. Initially, patients experience nyctalopia and peripheral vision loss, which progresses over time and may lead to complete vision loss. While research for cures is progressing, including gene therapy and stem cell transplantation, currently, there are no preventive or curative treatments on the market [1].

However, with advancing technology, there are ongoing innovations in prosthetic vision. The Argus II is the first FDA-approved device in Europe and the USA for severe or profound vision loss secondary to retinitis pigmentosa. The patient undergoes surgical implantation of a microarray onto the macula, which bypasses the diseased photoreceptors to directly stimulate preserved inner retina layers via epiretinal microelectrodes. Patients wear glasses equipped with a video camera that relays visual input signals to this microarray, which stimulates the inner retinal layers that relay the inputs to the optic nerve and visual pathway. There is a wide range in visual outcomes in patients, but many notice an overall improvement in functional vision [2].

Studies have so far demonstrated robust safety protocols in phase I and II trials with 70% of patients experiencing no major adverse effects. The most common adverse events include conjunctival dehiscence or erosion, hypotony, and presumed endophthalmitis [1, 3]. To the authors' knowledge, there has been no previously reported case of phthisis after implantation of the Argus II.

## 2. Case Presentation

This case presents a 61-year-old male known to the retina clinic with progressive, severe vision loss secondary to RP. The patient has a self-reported history of “experimental vitrectomy” OU in Cuba in 1996, CE/IOL in the right eye (OD) in 2006, and cataract surgery and intraocular lens implant (CE/IOL) in the left eye (OS) in 2010 followed by pars plana vitrectomy (PPV) and removal of retained lens material in 2014, after which the patient was doing well. However, the patient's VA continued to deteriorate to NLP OD and LP OS. Therefore, he elected to have the Argus II implantation, which was done in his better seeing left eye in December 2017.

The following is the procedure details: first, we placed the external electronic case superotemporally and the band was passed under the muscles and secured with Watzke's sleeve in the superonasal quadrant. In order to do so, a 360-degree conjunctival peritomy was performed. The sub-Tenon space was opened in a blunt fashion in all quadrants. The four rectus muscles were identified with the muscle hook and isolated with a 2-0 silk suture. The muscle capsule was cleaned off with Q-tips. Two partial thickness scleral flaps were created inferonasally and superonasally to secure the strip. Electronic case with 2.5 mm silicone strip was passed under the muscles and belts and secured with a silicone sleeve superonasally. The case was tested with a central processing unit under sterile conditions, was secured with two 5-0 mersilene sutures superotemporally and one 5-0 mersilene sutures inferotemporally. Silicone strip was held in inferonasal and superonasal quadrants 12 mm behind the limbus with one 6-0 mersilene. Second, a 23-gauge pars plana vitrectomy with peeling of the posterior hyaloid face was performed. The infusion cannula was inserted at 5 o'clock and confirmed visually to be properly located in vitreous cavity inferotemporally. Two 23-gauge sclerotomies were placed at 10 and 2 o'clock. A 25-gauge chandelier-light source was placed inferiorly with a trocar system. The light pipe and vitrector were inserted in the vitreous cavity. Then after core vitrectomy, the posterior hyaloid face was stained with Kenalog and detached. It was excised successfully up to the periphery. Third, we extended the sclerotomy and passed the electronic array and filament intravitreally by extending the sclerotomy 5 mm superotemporally with a sharp blade 4 mm behind the limbus. The case was tested again with a CPK unit under sterile conditions, and an electronic array with filament was passed through it into the vitreous cavity with silicone sleeve-coated forceps. The sclerotomy was then closed with 5-0 mersilene sutures at the edges of the filament. Next, we placed the microelectrode array and perform transretinal and transchoroidal tacking of the array by placing the array on the surface of the macula holding from the manipulating knob with Eckhardt's forceps. It was orientated 45 degrees correctly to the transverse plane of the macula with the left inferior corner at the edge of the optic nerve. At 4 o'clock, 19-gauge sclerotomy was fashioned with an MVR blade. Then, retinal tack was loaded in the tacker, and bimanually, tack was passed through retina and choroid and tacked onto the posterior sclera, while keeping the microarray plate in position. Then, the donor-processed scleral patch was fashioned and secured with 7-0 vicryl. Similar patch was used to cover the coil inferotemporally and secured with 7-0 vicryl. Finally, we closed all sclerotomies and peritomy with 8-0 nylon and 7-0 vicryl, respectively.

**Figure 1:**
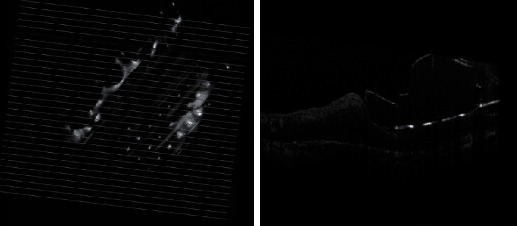
OCT image of the patient after Argus implantation.

The surgical implantation of the Argus II was successful, and the surgery was without complication (Figure 1). On postoperative day one, the patient's VA was LP OS, with elevated intraocular pressure (IOP) of thirty, which returned to normal one week later. At postoperative month one, the vision was stable at LP, with an IOP of seven. The eye was quiet, and the implant was working well; most importantly, the patient was satisfied with his ambulatory vision.

Four months after surgery, the patient presented complaining of aching pain; he was found to have preseptal cellulitis and was started on antibiotics. This cellulitis resolved over two weeks, but when the patient returned, he had a two mm hyphema associated with mild ocular inflammation without an inciting event or neovascularization on exam. The intraocular lens (IOL) was in the bag, and there was no reason for developing spontaneous hyphema. The hyphema was treated, and two weeks later, it had resolved without apparent incident. However, one month after the hyphema resolved, at postoperative month 6, the patient's VA OS diminished to NLP. In addition, the patient demonstrated phthisical changes, including hypotony (IOP 0 mmHg), Descemet membrane folds, and a vascular sheet posterior to capsular membrane. At no point in the patient's postoperative care, any posterior inflammatory or retinal issues were apparent, including retinal detachment or Argus dehiscence. There was no bleb formation or conjunctival dehiscence. Unfortunately, the phthisical progression affected the Argus II implant, which stopped transmitting the superior field.

The patient was started on steroids, but the eye remained hypotonus, and the patient's phthisical changes continued to progress despite therapy. Exploration of the device was offered to the patient, but the patient refused as it did not have any prognostic value in the setting of advanced retinitis pigmentosa.

## 3. Discussion

While there have been over 100 Argus II implants, there have been no reported cases of phthisis. In the Argus II clinical trial, the documented complications include hypotony, conjunctival erosion or dehiscence, presumed endophthalmitis, retacking, retinal detachment or tear, explants, device failures, uveitis, or corneal decompensation. Of note, most of these complications were clustered in the same patients, while 70% had no complications. It was also realized that the second half of patients had better outcomes likely due to improving surgical techniques [1, 3].

Phthisis is characterized by a shrinkage and disorganization of the globe, typically associated with a squared, opaque, and thickened cornea and sclera, NVI, and retinal detachments. Common causes include trauma, surgery, infection, inflammation, malignancy, retinal detachment, and vascular pathology, which lead to hypotony, inflammatory changes, or violation of the blood-ocular barriers. Pathologically, cells become disorganized and dysplastic. Early treatment of the cause is the only hope for possible cure, and phthisical eyes quickly become nonfunctional [4].

The etiology of the phthisical changes in this case can have several reasons such as fibrous downgrowth, ciliary shut down due to immune reaction, inflammation, or unreported trauma. It is uncertain why the patient would develop a spontaneous hyphema five months after surgery; it is possible the patient had unreported trauma that caused the hyphema and initiated the phthisical change. It was noted that at the time of the hyphema, the patient had a small inflammatory response, but that resolved within two weeks and was unlikely significant enough to induce enough of an immune response to cause phthisis.

**Figure 2:**
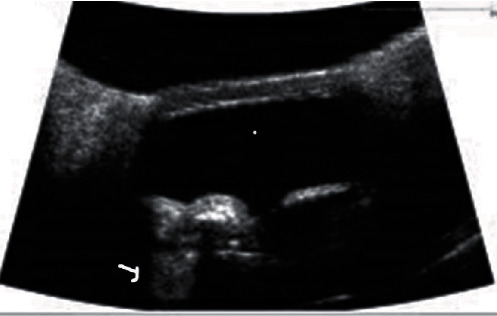
UBM image of the patient with hyperreflectivity in the ciliary body adjacent to the supraciliary space with a comet tail artifact (white arrow). It could be due to implant migration anteriorly and fibrous downgrowth.

The most probable etiology is that repeated posterior surgeries and the scleral wound for inserting the implant allowed fibrous downgrowth that could have led to phthisis (Figure 2). The patient had an “experimental vitrectomy” in 1996 in Cuba, another PPV for the removal of retained lens material in 2014, and implantation of the Argus II in 2017. It is uncertain what procedure was done in Cuba, but there were no foreign bodies found or issues with his eye before undergoing the third posterior segment surgery for implantation of the Argus II. Perhaps, the 5 mm pars plana sclerotomy used to implant the Argus microarray could have an increased risk of phthisis due to the large wound, and/or insertion of foreign material could stimulate fibrous downgrowth formation. Fibrous downgrowth has been reported by similar mechanisms, including after cases of traumatic corneoscleral wound dehiscence and telescope implantation [5, 6].

**Figure 3:**
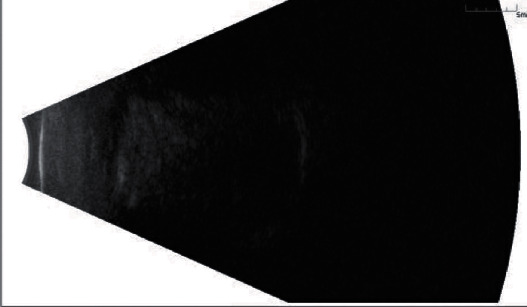
Echography of the patient shows intact retina.

The foreign material of the array consists of 60 platinum electrodes embedded in a polyimide, which is theoretically inert. There is also a cable which spans the eye wall to connect the microarray to the Argus II. While this is sutured closed, it is possible the exposed cable, which is composed of a metallized polymer, could lead to phthisis. The composition of the metallized polymer is not reported [1, 3], but it is another possibility that this exposed cable could be reactive or provide the environment for disorganization and phthisical change. The other reason for phthisis after any eye surgery can be due to retinal detachment, but that was ruled out with serial echography and examination (Figure 3).

## 4. Conclusion

While the cause of phthisis in this Argus II patient is not certain and may be multifactorial, it is important to note that phthisis is a possible complication of an Argus II implant, as this patient had no other obvious insult or reason for the phthisical change.

## Additional Points

*Authorship*. All authors attest that they meet the current ICMJE criteria for authorship.
